# Research advances in imaging markers for predicting hematoma expansion in intracerebral hemorrhage: a narrative review

**DOI:** 10.3389/fneur.2023.1176390

**Published:** 2023-04-25

**Authors:** Yong-Wei Huang, Hai-Lin Huang, Zong-Ping Li, Xiao-Shuang Yin

**Affiliations:** ^1^Department of Neurosurgery, Mianyang Central Hospital, School of Medicine, University of Electronic Science and Technology of China, Mianyang, Sichuan, China; ^2^Department of Immunology, Mianyang Central Hospital, School of Medicine, University of Electronic Science and Technology of China, Mianyang, Sichuan, China

**Keywords:** imaging markers, hematoma expansion, intracerebral hemorrhage, CT angiography, computed tomography

## Abstract

**Introduction:**

Stroke is a major global health concern and is ranked as the second leading cause of death worldwide, with the third highest incidence of disability. Intracerebral hemorrhage (ICH) is a devastating form of stroke that is responsible for a significant proportion of stroke-related morbidity and mortality worldwide. Hematoma expansion (HE), which occurs in up to one-third of ICH patients, is a strong predictor of poor prognosis and can be potentially preventable if high-risk patients are identified early. In this review, we provide a comprehensive summary of previous research in this area and highlight the potential use of imaging markers for future research studies.

**Recent advances:**

Imaging markers have been developed in recent years to aid in the early detection of HE and guide clinical decision-making. These markers have been found to be effective in predicting HE in ICH patients and include specific manifestations on Computed Tomography (CT) and CT Angiography (CTA), such as the spot sign, leakage sign, spot-tail sign, island sign, satellite sign, iodine sign, blend sign, swirl sign, black hole sign, and hypodensities. The use of imaging markers holds great promise for improving the management and outcomes of ICH patients.

**Conclusion:**

The management of ICH presents a significant challenge, and identifying high-risk patients for HE is crucial to improving outcomes. The use of imaging markers for HE prediction can aid in the rapid identification of such patients and may serve as potential targets for anti-HE therapies in the acute phase of ICH. Therefore, further research is needed to establish the reliability and validity of these markers in identifying high-risk patients and guiding appropriate treatment decisions.

## 1. Introduction

Stroke is a major global health concern and is ranked as the second leading cause of death worldwide, with the third highest incidence of disability (1). Intracerebral hemorrhage (ICH) is a type of stroke that occurs when a blood vessel ruptures within the brain, leading to bleeding and damage. It is a devastating condition, and unfortunately, the number of cases continues to rise each year, with an estimated 3.41 million new cases annually (1, 2). Previous studies have reported mortality rates of ICH was 30–50% at 30 days, with nearly 50% of patients dying within 2 weeks of symptom onset (3). The clinical outcome of ICH patients is significantly affected by the initial hematoma volume and location, with hematoma expansion (HE) being present in 33% of cases and serving as an independent predictor of poor clinical prognosis and secondary neurological deterioration (4). Although no unified diagnostic standard currently exists, Computed Tomography (CT) and CT Angiography (CTA) examination are commonly used. Therefore, investigating the baseline CT scan and CTA images of ICH patients is critical for efficient clinical management. In recent years, many studies have identified specific CTA, CT and magnetic resonance imaging (MRI) manifestations that are associated with HE, including the spot sign (SpS) (5), leakage sign (LS) (6), spot-tail sign (STS) (7), iodine sign (IoS) (8), island sign (IS) (9), satellite sign (SaS) (10), blend sign (BS) (11), swirl sign (SwS) (12), black hole sign (BHS) (13), hypodensities (14), fluid-blood level (FBL) (15), subarachnoid extension (SAHE) (16), and MRI SpS (17). These imaging markers provide a more powerful approach for identifying patients at high risk of HE. This review aims to explore the potential correlation between specific hematoma manifestations on CTA and CT and early HE, with the goal of timely and effectively intervening in ICH patients with HE and reducing mortality rates while improving outcomes.

## 2. The definition of HE

In most studies, HE is defined as an increase in volume of the hematoma greater than 12.5 ml or more than 33% compared to the initial CT scan in follow-up CT scans (2). However, for studies involving CTA contrast agent extravasation, HE is defined as a proportional increase of 33% or an absolute increase in hematoma volume of more than 6 ml (5, 18, 19). Despite the frequent use of the definition of HE as >33% or >12.5 ml in recent studies, a consensus on the definition of clinically significant HE has not yet been reached. It is essential to establish an optimal definition of HE, both mathematically and clinically, to reduce heterogeneity among studies and to better stratify high-risk patients with ICH. Baseline CT scans are typically performed within 6 h of symptom onset, and follow-up CT scans are performed within 24 h after the baseline scan. Methods for calculating hematoma volume include the ABC/2 (Coniglobus formula), area measurement, and three-dimensional drawing. Semi-automatic measurement technology is more accurate than the ABC/2 formula, particularly for irregularly shaped hematomas (20). However, the Coniglobus formula has a maximum error of up to 20% in the calculation of irregular hematomas, which is extremely unfavorable for evaluating the patient’s condition with ICH. In recent years, a few studies have utilized medical software to calculate the initial hematoma volume (21, 22). For example, the 3D Slicer software (Version 4.8.0, Harvard University, NY) has a very small error in the calculation of hematoma volume and can detect inapparent HE. Therefore, the use of 3D Slicer for calculating hematoma volume is more accurate and can improve the assessment of patients’ condition.

## 3. Etiology

**Figure 1:**
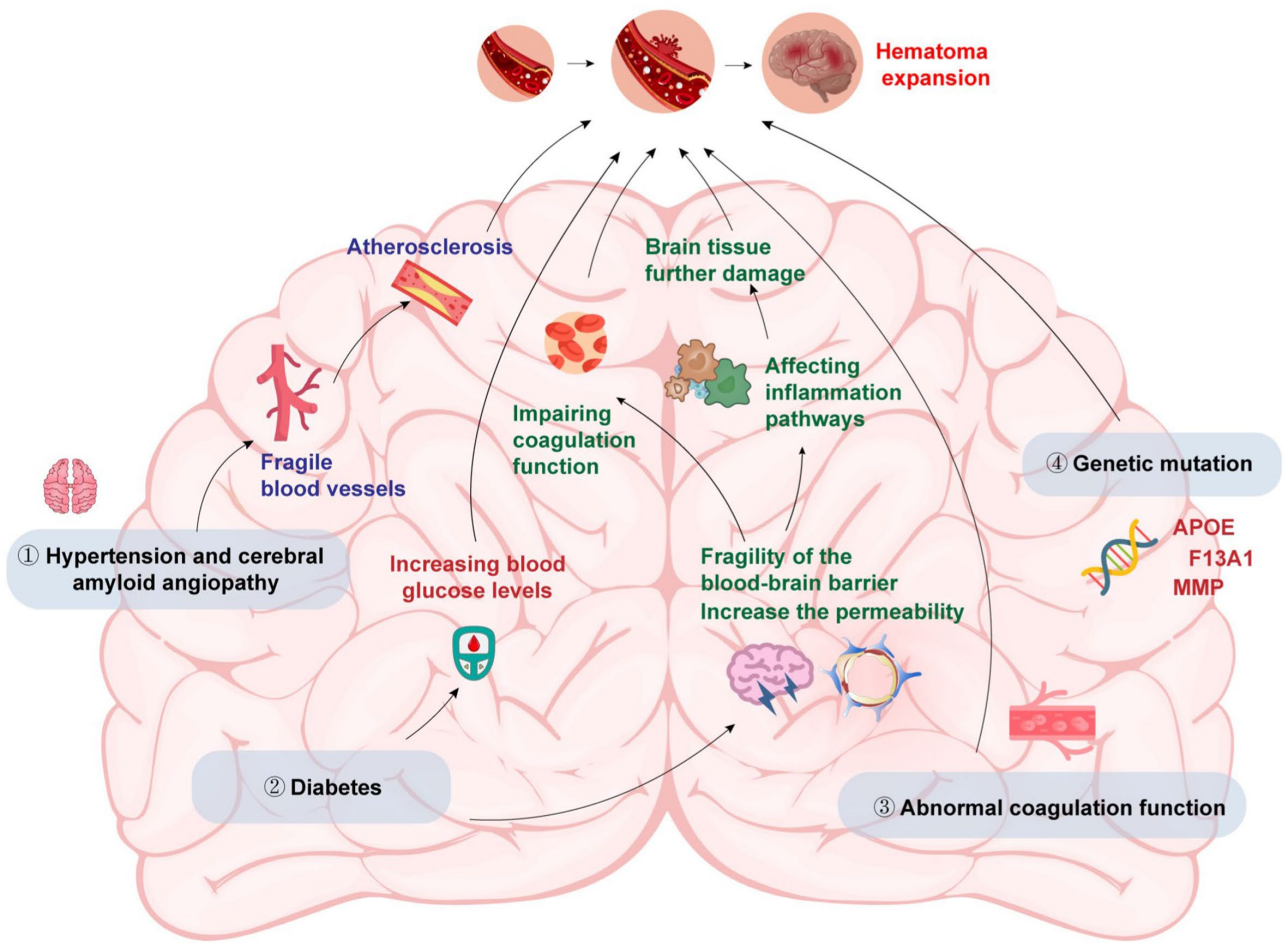
The etiology of hematoma expansion in intracerebral hemorrhage (by Figdraw).

The main factors that cause HE in patients with ICH include primary hypertension & cerebral amyloid angiopathy (CAA), diabetes, abnormal coagulation and genetic variations (Figure 1).

### 3.1. Primary hypertension and CAA

Both of them have the potential to cause blood vessel wall thickening, arteriosclerosis, and fragile blood vessels, increasing the risk of HE (23). Early studies showed that anti-hypertensive treatment could efficiently prevent HE, but subsequent studies found that there was no significant correlation between anti-hypertensive treatment and limiting HE and reducing mortality (24). The reason may be that before admission, the HE had already happened or HE may occur a few minutes after the onset of ICH. So anti-hypertensive treatment after admission did not improve the patients’ condition.

### 3.2. Diabetes

Liu et al. (25) found that early HE was associated with high osmotic pressure caused by elevated blood sugar. Elevated blood glucose levels can increase blood–brain barrier (BBB) permeability and lead to its disruption, making it easier for blood components to enter the brain tissue and cause further damage. The disruption of the BBB may also facilitate the infiltration of inflammatory cells into the brain, which can exacerbate the inflammatory response and contribute to HE. Besides, diabetes can impair coagulation function and alter the activity of several coagulation factors, resulting in a procoagulant state that favors the formation of blood clots. When a vessel ruptures in the brain, the formation of blood clots can contribute to the formation of the initial hematoma. Additionally, the procoagulant state may also contribute to the enlargement of the hematoma by promoting the formation of microthrombi that occlude small vessels and lead to ischemic damage in the surrounding tissue. Finally, diabetes can also lead to the activation of various inflammatory pathways, including the nuclear factor-kappa B pathway, which can promote the production of pro-inflammatory cytokines and increase oxidative stress in the brain tissue. These effects can contribute to the progression of the initial injury and exacerbate HE.

### 3.3. Abnormal coagulation

Abnormal coagulation could significantly increase the risk of HE. Patients with ICH were often accompanied by abnormal blood coagulation, and anticoagulant drugs were used, especially anti-platelet aggregation drugs (26). Restoring coagulation function could strongly reduce the risk of HE and improve the outcome.

### 3.4. Genetic variations

Studies have shown that genetic variations in certain genes are associated with an increased risk of HE and poor clinical outcomes in patients with ICH. Several single-nucleotide polymorphisms in the apolipoprotein E (APOE) gene have been identified as risk factors for HE and poor outcomes in ICH patients (27). APOE plays a key role in lipid metabolism and transport in the brain. In ICH, the APOE gene may also influence the metabolism and clearance of blood in the brain, leading to an increased risk of HE. Other genes that have been linked to HE in ICH patients include the factor XIII subunit A gene, which is involved in blood coagulation and clot stability, and the matrix metalloproteinase gene family, which is involved in the breakdown of the extracellular matrix and tissue remodeling (28, 29). Variations in these genes may lead to impaired coagulation and clot stability, or increased breakdown of the extracellular matrix, which can promote HE and poor outcomes in ICH. Furthermore, genetic factors may also interact with other clinical and environmental factors to increase the risk of HE. For example, a study found that the combination of certain genetic variants and high blood pressure was associated with an increased risk of HE in ICH patients (30). Another study found that the interaction between genetic variants and smoking was associated with an increased risk of HE and poor outcomes in ICH patients (31).

## 4. Pathophysiology

According to pathological evidence, the enlargement of hematoma may be attributed to secondary mechanical shearing of the adjacent vessels at the initial site of bleeding. In the 1970s, Fisher et al. (32) proposed the “avalanche” model to explain how primary ICH caused secondary mechanical damage to adjacent vessels. Subsequent studies have revealed that the volume of hematoma in patients with ICH is bimodal, and whether it is a micro-hematoma or a large hematoma is consistent with the “avalanche” injury process (33). According to the “avalanche” model, the enlarged part of the hematoma corresponds to the site of the “SpS” which indicates active bleeding in CTA. Multiple “SpSs” in the same hematoma suggest that multiple blood vessels are bleeding simultaneously (34). Some researchers believed that a single vessel rupture and persistent bleeding caused HE in patients with ICH (35), but so far, there is no direct pathophysiological evidence to support this theory. However, recent studies have shown that vascular abnormalities, including microaneurysms, exist in the brain tissue surrounding the hematoma and are likely to play a role in the pathogenesis of HE (36, 37). Moreover, studies have also suggested that coagulation abnormalities and inflammation may contribute to the development of HE (38, 39). Furthermore, the activation of the inflammatory cascade, generation of coagulation end-products, and hemoglobin degradation products lead to the initiation of a secondary injury cascade, which proceeds *via* diverse molecular pathways, including but not limited to mitochondrial failure, iron-mediated oxidative stress, and sodium accumulation. Ultimately, these mechanisms result in the generation of proinflammatory mediators that trigger the breakdown of the blood–brain barrier, cerebral edema, and neuronal apoptosis (40). Therefore, it is imperative to conduct further preclinical and clinical research to gain deeper insights into the pathophysiology of both HE and ICH and identify potential therapeutic targets to prevent or minimize its development.

## 5. Outcome

Recent studies have further confirmed the strong association between HE and secondary neurological deterioration, poor clinical outcomes, and mortality in patients with ICH (4, 41). A dose–response relationship has been observed, indicating that for every 10% increase in hematoma volume, the case fatality rate increases by 5%, and for every 1 ml increase in hematoma volume, the likelihood of ICH patients transition from independent living to being unable to care for themselves increases by 7% based on modified Rankin scale (mRS) evaluations (42). Moreover, the degree of HE expansion has been consistently related to functional prognosis and mortality, regardless of the definition of HE. These findings suggest that identifying patients at high risk of HE and implementing targeted interventions to prevent HE and its sequelae may lead to improved clinical outcomes in patients with ICH.

Moreover, the impact of HE on patient outcomes is not limited to immediate mortality and disability. Studies have shown that HE is also associated with long-term functional and cognitive impairment, reduced quality of life, and increased risk of recurrent ICH (43, 44). The underlying mechanisms of these long-term consequences are not fully understood but may be related to ongoing neuroinflammation and secondary injury to surrounding brain tissue.

To mitigate the impact of HE, early identification of patients at high risk of HE is crucial. In addition to hematoma volume, other imaging markers such as BS, BHS, and IS have also been proposed as predictors of HE (45, 46). However, these markers require specialized training and may not be widely available. Recently, machine learning algorithms have been applied to automatically identify these markers and predict HE, showing promising results (47, 48). Overall, while HE is a common and serious complication in patients with ICH, the development of reliable and accessible imaging markers and targeted interventions may help to improve patient outcomes and reduce the burden of this devastating disease.

## 6. Characteristic imaging markers of HE

Based on the different imaging technique, we narrate the imaging markers on CTA, CT, and MRI, respectively. A summary of the imaging markers associated with HE in ICH is presented in Table 1.

**Table 1:** Summary of imaging markers associated with HE in ICH.

Imaging Markers		Author	Year	Nation	Study Design	Paticipants (*n*)	Male (%)	Age (y)	Positive imaging markers (*n*)	Primary outcome	SEN (%)	SPE (%)	PPV (%)	NPV (%)
CTA	Spot Sign	Wada et al. (5)	2007	Canada	prospective observational multi-center	39	74.29	64 (31–85)	13	HE	91 (62–100)	89 (72–96)	77 (50–92)	96 (81–99)
	Leakage Sign	Orito et al. (6)	2016	Japan	prospective single-center	80	47.5	67.9 (44–93)	35	HE	93.3 (75.7–98.8)	88.9 (81.5–91.2)	—	—
	Spot-Tail Sign	Sorimachi et al. (7)	2013	Japan	retrospective single-center	141	—	64.3 ± 13.1	15	Acute deterioration	—	—	—	—
	Iodine Sign	Fu et al. (8)	2018	China	prospective single-center	91	70.33	53.82 ± 12.83	52	HE	91.5	79.5	82.7	89.7
										Poor outcome	61.5	94.9	94.1	64.9
CT	Island Sign	Li et al. (9)	2017	China	retrospective single-center	252	66.27	IsS (+): 62.3 ± 11.1 IsS (−): 59.2 ± 12.2	41	HE	44.7	98.2	92.7	77.7
	Satellite Sign	Shimoda (10)	2017	Japan	retrospective single-center	241	50.21	70.3 ± 13.0	98	Poor outcome	54	94	95.9	44.1
	Blend Sign	Li et al. (11)	2015	China	retrospective single-center	172	68.02	BS (+): 60.21 ± 12.5 BS (−): 60.45 ± 11.9	29	HE	39.3	95.5	82.7	74.1
	Swirl Sign	Selariu (49)	2012	Sweden	retrospective single-center	203	44.83	73.0 ± 14.0	61	Poor outcome, mortality	—	—	—	—
	Black Hole Sign	Li et al. (13)	2016	China	prospective single-center	206	65.53	60.3 ± 12.2	30	HE	31.9	94.1	73.3	73.2
	Hypodensities	Boulouis et al. (14)	2016	U.S.	prospective cohort single-center	1,029	45.09	71.8 ± 12.7	321	HE	62	77	40	89
	Subarachnoid Extension	Morotti et al. (16)	2020	Italy	retrospective single-center	552	54.71	Development Cohort: 70 (61–77)	147	HE, mortality	83 (71–92)	56 (45–67)	57 (45–67)	83 (70–91)
								Replication Cohort: 77 (66–83)			73 (54–87)	60 (45–73)	52 (37–68)	79 (62–90)
MRI	Spot Sign	Valyraki et al. (17)	2023	France	retrospective single-center	147	65.99	66 (53–80)	91	HE	90 (74–98)	47 (37–58)	94 (83–99)	35 (25–46)

### 6.1. Imaging markers on CTA

#### 6.1.1. Spot sign

**Figure 2:**
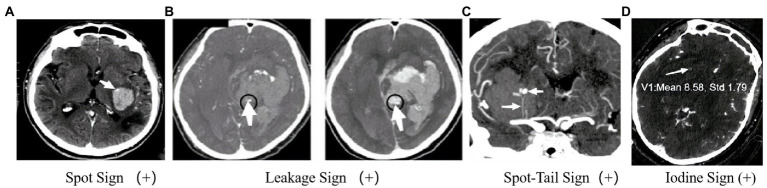
Imagination markers on CTA. **(A)** Enhanced focus within a hematoma indicates contrast agent extravasation and reveals the site of vessel rupture (5); **(B)** In the CTA phase, an enhanced focus within the hematoma is observed, and further observation in the delayed CTA phase can identify an increase in CT value within a specific region of interest. A larger enhanced focus may indicate persistent bleeding and HE (6). **(C)** The presence of an intrahematoma striate artery and an enhanced focus within the hematoma may suggest an arterial rupture and pinpoint the location of bleeding (7). **(D)** In GSI, an internal focus with an IC >7.82100 μg/ml indicates a positive IoS (8).

The SpS presented on the CTA referred to the “enhanced focus in the hematoma” in the original image (5) (Figure 2A). The biological basis of the SpS is not yet fully understood, but recent studies suggest that it may be related to increased permeability of cerebral vessels, indicating a higher risk of HE. According to precious study, the permeability of CT perfusion imaging (CTP) could identify whether there is a SpS. The permeability refers to the rate of contrast agent overflowing from the cerebral vascular, the higher the permeability, the greater the possibility of HE (50). At present, early clinical manifestations, coagulation, APOEε2 alleles, Glasgow Coma Scale at onset, mean arterial pressure > 120 mmHg, and intraventricular hemorrhage (IVH) were associated with the appearance of the SpS (51) have validated the reliability of SpS as an independent predictor of HE in patients with ICH (51, 52). Demchuk et al. (18) have reported that the sensitivity (SEN), specificity (SPE), positive predictive value (PPV) and negative predictive value (NPV) of the SpS for HE were 51.00, 85.00, 61.00, and 78.00%, respectively. Delgado et al. (51) proposed a grading system for quantization of SpS, including number of spot sign, maximum attenuation (H), and maximum size. And studies have shown that the grading system of SpS could not only predict the early incidence of HE in ICH patients, but also accurate classification of HE, in-hospital mortality and clinical outcome (53). This will help to further screen high-risk ICH patients.

Recent research has focused on refining the use of the SpS for clinical decision-making in ICH patients. For example, a recent study found that incorporating the SpS and other clinical factors into a predictive model could accurately identify patients at high risk of HE and guide treatment decisions (54). Other studies have explored the use of machine learning algorithms to automatically detect and quantify the spot sign, which may improve the efficiency and accuracy of diagnosis (55, 56). Overall, the SpS is a promising imaging marker for predicting the risk of HE in ICH patients, and its accurate detection and quantification may help to guide clinical decision-making and improve patient outcomes. However, further research is needed to fully understand the underlying biological mechanisms of the SpS and to refine its use in clinical practice.

#### 6.1.2. Leakage sign

In 2016, Orito et al. (6) proposed the concept of LS based on previous studies on SpS and established a method to determine the positive LS based on the comparison of CTA phase and delayed CTA phase images (Figure 2B). Firstly, each evaluator was asked to set a region of interest (ROI) with a 1 cm margin on the delayed CTA phase image, which was considered the area with the highest HU change between the CTA phase and delayed CTA phase. Secondly, the same ROI was then placed on the CTA image in the same anatomical area. The HU values in the ROI of the CTA phase and delayed CTA phase images are calculated, and an HU increase >10% was considered the positive LS, indicating subtle contrast agent extravasation. Their study found that the LS had a higher SEN (93.30%) and SPE (88.80%) for predicting HE compared to the SpS. Furthermore, patients with positive LS had a significantly worse prognosis than those with negative LS. In fact, the LS may represent a dynamic change of the hematoma and be a more sensitive marker for predicting HE in ICH patients. However, further research is needed to validate these results and establish the clinical utility of the LS. Another potential issue is that this method may result in higher radiation exposure. However, if HE can be diagnosed, the clinical benefits outweigh the additional radiation exposure risk. The presence or absence of the LS does not significantly affect surgical indications. Using the LS to predict HE may help identify patients who need early hematoma evacuation surgery. In addition to surgical indications and aggressive treatment, this method can help understand the dynamic changes in ICH in clinical medicine.

#### 6.1.3. Spot-tail sign

The STS, proposed by Sorimachi et al. (7) in 2013, combines the SpS with the presence of an intrahematoma striate artery on CTA coronal image (Figure 2C), and has shown potential as a more accurate predictor of HE and acute neurological deterioration compared to the SpS alone. Recent studies have further supported the usefulness of the STS. For example, a study by Phan et al. (57) found that the STS was associated with a higher risk of early neurological deterioration, and was an independent predictor of HE, while the SpS alone was not significant in predicting these outcomes. Another study by Li et al. (58) found that the presence of the STS was associated with larger hematoma volume, more frequent IVH, and worse clinical outcome.

One possible explanation for the association between the STS and HE is that the striate artery represents the site of active bleeding, and the sustained blood supply through the striatum to the bleeding site promotes HE. This hypothesis is supported by angiographic images showing contrast agent extravasation from the striate artery. In conclusion, if the hypothesis is valid (i.e., the striate artery is the location of active bleeding), the STS may be a more sensitive predictor of HE and acute neurological deterioration when compared to the SpS. However, more research is needed to confirm this hypothesis and further clarify the mechanism of HE.

#### 6.1.4. Iodine sign

**Figure 3:**
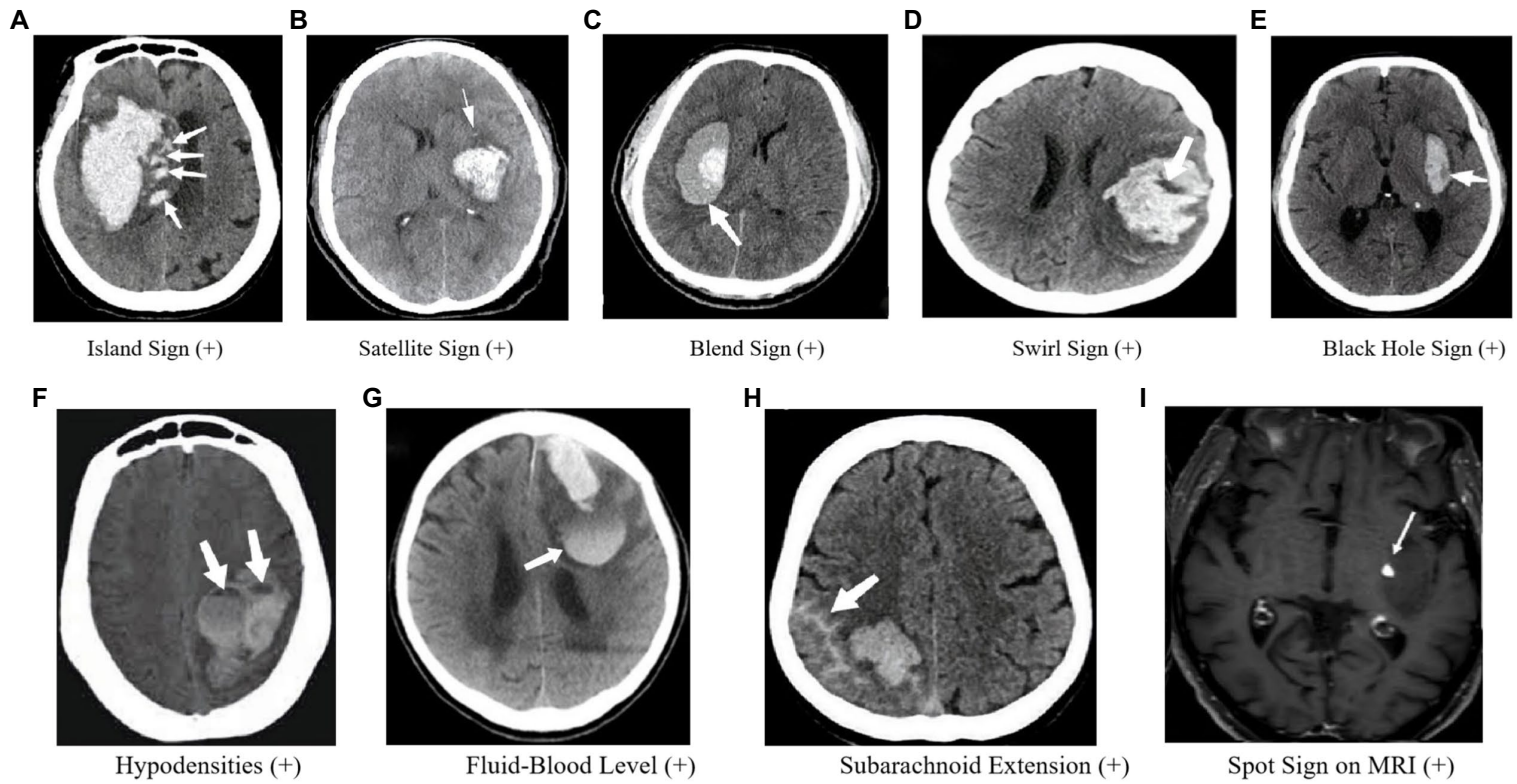
Imaging markers on CT and MRI. **(A)** Four or more small hematomas, some of which may be separate from the main hematoma, may indicate persistent bleeding from small vessels in the surrounding area, which could lead to HE. **(B)** A small hematoma located around the main hematoma, referred to as “satellites” (10); If the “satellite” develops further, it may become IS. So the SaS may represent a dynamic change of IS. **(C)** A relatively low-density region with an adjacent high-density region within the hematoma and a well-defined margin between these two regions. **(D)** Low-density region within a high-density hematoma (59). **(E)** Low-density area within the hematoma that is completely surrounded by adjacent high-density hematoma (13). **(F)** Hypoattenuation is associated with the hyperattenuated hematoma with a well-defined density difference between the two attenuation regions (14). **(G)** A horizontal interface between hypodense bloody serum and hyperdense fluid (15). **(H)** SAHE may represent the fragility of vessels as well as the active bleeding in the hematoma surrounding the vessels, lending credence to the “avalanche” hypothesis (16). **(I)** One spot was found on contrastenhanced T1-weighted sequence (17).

Gemstone spectral imaging (GSI) is a promising scanning mode that enables direct separation of iodine from the blood and subsequent reflection of iodine concentration (IC) by monochromatic imaging. Consequently, a novel method called the IoS has been introduced (Figure 3C), which allows for direct reflection of leaking iodinated contrast and prediction of HE. A positive IoS was defined as: (1) ≥1 enhanced foci on the iodine-based decomposition image within the hematoma of any size and morphology, assessed by visual inspection (conducted by nonradiologists); (2) an internal focus IC >7.82100 μg/ml, measured by reviewers using a region of interest that covered most of the focus area (magnified from ×3 to ×5); (3) discontinuity from adjacent normal or abnormal vasculature. A study conducted by Fu et al. (8) demonstrated that the IoS was a reliable and sensitive marker for predicting HE and poor functional outcomes in ICH patients. Another comparative study of BHS, SaS, and IoS in predict HE in patients with spontaneous ICH demonstrated that the presence of GSI-based IoS had a better predictive value for HE with higher sensitivity and accuracy (60). Despite the usefulness of spectral imaging, its availability and application may not be feasible in various medical institutions, thereby potentially limiting the prevalence of the IoS. Further large-sample and multi-center studies were still urgently needed to identify whether the IoS is a reliable and sensitive marker for predicting HE and poor functional outcomes or not.

### 6.2. Imaging markers on CT

#### 6.2.1. Shape-related imaging markers

##### 6.2.1.1. Island sign

In 2017, Li et al. (9) proposed the IS as an independent predictor of HE and poor functional outcome in patients with ICH (Figure 3A). The sign is defined as the presence of three or more small hematomas scattered and separated from the main hematoma or four or more small hematomas, some of which are separated from the main hematoma. The island hematoma is round or oval and separate from the main hematoma, and the small hematoma associated with the main hematoma should be vesicular or budlike but not lobulated. The IS represents extreme margin irregularities and is a refinement of the shape irregularity scale. Huang et al. (21) further suggested that the IS is a strong predictor of HE and is useful for scoring the prediction of HE in ICH. In comparison, Zheng et al. (61) showed that although the accuracy of the IS for predicting HE is lower than the SpS, it can be an alternative predictor if CTA cannot be performed. Moreover, Zhang et al. (62, 63) validated Li et al.’s findings and showed that the IS predicts both early HE and long-term poor clinical prognosis, and admission serum glucose is associated with HE and IS. Huang et al. (22) also observed that the incidence of the IS was higher in patients with larger hematomas, implying that “worse hematomas get worse.” However, further research is required to explore the pathogenesis of the IS in larger hematomas. Nonetheless, the IS is a useful imaging marker that can aid in predicting HE and poor clinical outcomes in patients with ICH.

##### 6.2.1.2. Satellite sign

In 2017, Shimoda et al. (10) proposed the definition of the SaS (Figure 3B), which is characterized by a small hematoma completely separated from the main hematoma, with a diameter smaller than 10 mm and a distance between the small and main hematoma ranging from 1 to 20 mm. A study of 257 patients with sICH showed that the presence of at least one SaS in CT images was an independent and serious risk factor for poor prognosis in patients with sICH (10). However, the relationship between SaSs and HE is still unclear. The SaS was found to be strongly associated with increased blood pressure, decreased activated partial thromboplastin time, large hematoma volume, and IVH at admission, which may help predict the prognosis of patients with sICH. The authors suggested that metabolic changes occurring around the hematoma are associated with cytotoxic effects, which may lead to hemorrhagic transformation or reperfusion injury, ultimately resulting in the destruction of the capillary blood–brain barrier and the formation of a SaS as a lesion around the hematoma. However, further research is needed to determine the mechanism of hemorrhage around the hematoma.

It is important to note that some SaSs may actually be part of an irregular hematoma, which is thought to be the result of multiple arteriolar hemorrhage (49, 64–66). Barras et al. (64) identified the SaS as a part of the cut end of a lobulated irregular hemorrhage. Despite this, the SaS can still be used as a predictor for patient outcomes due to its clear definition and easy identification. A comparative study of SaS and SpS in 153 patients with sICH found that the SaS is an independent predictor for HE, with a SEN and SPE of 59.46 and 68.97%, respectively (67). Although the SpS has higher predictive accuracy, the SaS is an acceptable predictor for HE when CTA is unavailable. The incidence of HE in patients with supratentorial hemorrhage is higher in those with positive SaSs compared to irregular hematoma. Therefore, the SaS is a simple image marker that has proven to be of acceptable predictive value for HE. However, further research is needed to verify the underlying mechanisms of the SaS.

#### 6.2.2. Density-related imaging markers

##### 6.2.2.1. Blend sign

In some institutions, the use of CTA examination to assess ICH patients may be limited. As a result, researchers have explored other imaging markers that can predict HE on CT scans. In 2015, Li et al. (11) proposed the BS as a potential marker (Figure 3D). The BS is characterized by a relatively low-density region with an adjacent high-density region within the hematoma, with a well-defined margin between these two regions. The difference in Hounsfield units between these two regions should be at least 18 Hu, and the relatively low-density region should not be completely surrounded by the high-density region.

In a study of 172 ICH patients, the BS was detected in 29 (16.9%) patients on the baseline CT scan. The SEN, SPE, PPV, and NPV of the BS for predicting HE were 39.30, 95.50, 82.70, and 74.10%, respectively. The specificity of the BS was found to be higher than that of the SpS. The baseline hematoma volume of patients with a positive BS was larger than that of patients with a negative blend sign. Additionally, the hematoma was more likely to expand in patients with a positive BS, suggesting that the BS could be used as an independent predictor of HE.

The BS reflects the attenuation of hematoma density on CT scans in patients with different stages of ICH (68). The density of the hematoma is affected by its composition, with hemoglobin being an important factor in determining its appearance on CT scans. As blood coagulates, the hematoma shows high density on CT scans. On the other hand, when there is active bleeding, the hematoma tends to be lower in density than blood clot condensate. The appearance of the BS is due to the presence of mixed blood at different bleeding times, and hematoma re-bleeding can further lead to HE (11).

##### 6.2.2.2. Swirl sign

The SwS is an imaging finding observed in intracranial hyperattenuated hematomas (Figure 3E), which refers to region(s) of hypoattenuation or isoattenuation within the hyperattenuated ICH that can vary in shape and be rounded, streak-like or irregular (12, 68). Its definition has evolved over time, with Selariu et al. (59) in 2012 defining it as areas of hypoattenuation or isoattenuation compared to brain parenchyma, observed on both axial and coronal planes. The prognostic value of the SwS in spontaneous ICH has been explored in several studies. Kim et al. (69) in 2008 found a univariable association between the SwS and poor clinical outcome, but no association with HE. However, Selariu et al. (59) later reported that SwS were less prevalent in smaller hemorrhages, indirectly suggesting a lower risk of HE. Ng et al. (70) and Huang et al. (21) both found an association between the SwS and HE. In a comparative study of the black hole sign (BHS) and SwS (71), the SEN, SPE, PPV, and NPV of the SwS for predicting HE were 46.50, 71.30, 47.00, and 71.00%, respectively, and multivariate logistic regression showed that the presence of SwS on admission CT does not independently predict HE in patients with ICH. Therefore, further research is necessary to determine the true prognostic value of the SwS in HE.

##### 6.2.2.3. Black hole sign

In recent years, researchers have identified a phenomenon called the BHS on CT scans of patients with ICH (Figure 3F). The BHS is characterized by a low-density area within the hematoma that is completely surrounded by adjacent high-density hematoma. The sign has a clear boundary, is not connected to adjacent brain tissues, and the CT values of the two density regions within the hematoma differ by at least 28 HU. Studies have shown that the BHS is a good predictor of early HE. In a study of 206 ICH patients, 30 (14.6%) were found to have the BHS on their baseline CT scans. The SEN, SPE, PPV, and NPV of predicting early HE were 31.90, 94.10, 73.30, and 73.20%, respectively (13). In a comparative study of the BHS and another sign called the BS both were found to be good predictors of HE, with the BS showing a slightly higher level of accuracy (72). In another investigation of 129 ICH patients, both the SpS and BHS appeared to have good predictive value for HE, but the SpS seemed to be a better predictor (73). Furthermore, the presence of the BHS on initial CT scans independently predicted poor clinical outcomes at 90 days, according to a study of 225 patients (74). The authors found that patients with the BHS were significantly more likely to have a poor clinical outcome (defined as mRS ≥ 4) than those without (84.4% vs. 32.1%). Hematoma heterogeneity has also been shown to be associated with HE (52). However, assessing heterogeneity is subjective, and there is no established and reliable imaging standard for its assessment. The appearance of the BHS suggests that there are bleeding episodes at different periods within the heterogeneous hematoma and could be a useful predictor of HE in patients with ICH.

##### 6.2.2.4. Hypodensities

Studies have demonstrated a correlation between hypodensities and HE following ICH (14, 75) (Figure 3G). Moreover, unsatisfactory outcomes at 90 days have been linked to hypodensities. Factors such as a larger hematoma size, prior anticoagulation use, the SpS on CTA, and a shorter time to CT have been associated with hypodensities (76). In one study, the optimal detection time for hypodensities was 1.5–3 h with a cut-off point of 114.5 min. Therefore, vigilance is advised for clinicians when hypodensities are detected between 1.5 and 3 h after ICH onset to prevent secondary neurological deterioration (77).

##### 6.2.2.5. Fluid-blood level

In patients with ICH, baseline CT scans have occasionally revealed FBL (15, 78–81) (Figure 3H), which is defined as a horizontal interface between hypodense bloody serum and hyperdense fluid that has settled dorsally and is visible on CT scans (15, 79). The presence of FBL on Non-Contrast Computed Tomography (NCCT) scans has been linked to anti-coagulation use, a lobar location, and an increased risk of HE (79, 80). As a result of hematoma liquefaction, hemorrhage extravasation into pre-existing cystic cavities leads to FBL (15, 81). A recent study has also suggested that FBLs may serve as a vital marker of HE in patients with ICH associated with CAA (15).

The density of the hematoma on NCCT may potentially suggest distinct phases of bleeding and may be connected with clinical progression following symptom onset. NCCT attenuation is time-independent in ICH, and hematoma density fluctuation is related to clot development and the sedimentation of cellular components in the plasma. The content of hemoglobin primarily determines density on NCCT, with protein-rich plasma appearing hypodense on NCCT in the initial phase of ICH relative to surrounding tissue (82). Clot retraction causes a relative hyperattenuation on NCCT, leading to heterogeneity in hematoma density, which may serve as a valuable predictor of the risk of HE or a poor outcome (83, 84).

#### 6.2.3. Subarachnoid extension

SAHE, a new imaging marker for predicting HE and poor functional outcomes in ICH patients, was recently proposed (16) (Figure 3I). The researchers discovered that SAHE could predict HE in individuals with lobar ICH. SAHE was observed to occur in 27.8% of the development cohort and 24.5% of the replication cohort. A multivariate study demonstrated that SAHE independently predicted the probability of HE in patients with lobar ICH after controlling for confounding variables. SAHE may show the existence of weak vessels as well as active bleeding in the hematoma surrounding the vessels, lending credence to the “avalanche” hypothesis of HE (15). Furthermore, earlier research has indicated that the presence of cortical superficial siderosis on MRI was substantially linked with a higher volume in individuals with lobar ICH, supporting this assumption indirectly (85–87).

### 6.3. Imaging markers on MRI

#### 6.3.1. Spot sign

The identification of SpS on MRI was first proposed by Muran et al. (88) in 1998. At that time, the concept of SpS did not exist, and any high-intensity signals on T1-weighted post-contrast images were thought to be due to extravasation of contrast medium. In a study of 108 patients, extravasation was observed in 39 patient. Extravasation on MRI was found to be closely correlated with HE, indicating ongoing bleeding. Aviv et al. (89) later developed an animal model of contrast extravasation (SpS) in acute ICH based on MRI, but no significant correlation was found between SpS and HE. Since there was no corresponding MRI marker for SpS at the time, Katharina et al. (90) conducted further research and found that SpS could be detected using post-contrast T1-weighted and dynamic T1-weighted MRI images. The presence of SpS on MRI was found to be associated with worse clinical outcomes, and the time course of contrast extravasation in dynamic T1 images indicated ongoing bleeding. These findings were consistent with those of Muran et al. (88). Valyraki et al. (17) then defined SpS on MRI for the first time, with the following criteria: (1) spot-like or serpiginous high signal intensity >1.5 mm in at least one dimension, located within the margin of the hematoma and without connection to an outside vessel; and (2) no hyperintensity at the corresponding location on non-enhanced T1-weighted time-of-flight magnetic resonance angiography (MRA). In a study of 147 patients, the presence of SpS on MRI was found to be an independent biomarker of HE, and the presence of ≥2 spots was independently associated with a poor 3-month outcome. Conversely, the lack of SpS was highly predictive of a favorable evolution. Due to a corresponding MRI marker is lacking to date, further investigation of imaging markers on MRI is urgent for identifying ICH patients with high risk of HE.

## 7. Minimal computed tomography attenuation value

Chu et al. (91) discovered that the MCTAV is an independent predictor of HE and poor functional outcomes. In their study, the MCTAV was measured by manually selecting a region of interest before the software automatically calculated the CT minimal attenuation value. Their results revealed high SEN (64%) and SPE (92%) for identifying patients at risk of HE.

## 8. Application of imaging markers and suggestions for clinicians

Using imaging markers and patient clinical information to develop scoring systems or nomogram models to identify high-risk HE patients is a clinical method for risk stratification and further predicting functional outcome. Different medical institutions may have varying medical conditions, so it is necessary to choose appropriate imaging markers to predict the risk of HE based on reality. In 2018, Morotti et al. (92) proposed the BAT score, which includes an easy-to-use 5-point prediction score, including positive BS (1 point), any hypodensity (2 points), and time from onset to NCCT <2.5 h (2 points). Their findings showed that the BAT score can identify subjects at high risk of HE with good specificity and accuracy. This tool requires just a baseline NCCT scan and may help clinicians in poor medical institutions distinguish high-risk HE patients. In 2020, Fu et al. (93) proposed a 10-point prediction score, including baseline ICH volume ˃ 30 ml (1 point), time to initial CT scan ≤3 h (2 points), IS (6 points), and BHS (1 point). They demonstrated that the SEN, SPE, PPV, and NPV of the score ≥ 3 for predicting HE were 97.8, 92.7, 90.9, and 98.3% with high accuracy. Similarly, Huang et al. (21) developed a 7-point prediction score, including hours from onset to CT ≤ 6 h (1 point), baseline ICH volume ˃ 30 ml (1 point), positive IS (1 point), positive BS (1 point), positive SwS (1 point), anticoagulant use or an INR > 1.5 (1 point), and IVH extension (1 point). They indicated that the score system had reliable accuracy in predicting HE, and NCCT imaging markers may serve as the key for HE prediction. Yang et al. (94) put forward a new prediction models of functional outcome in acute ICH patients, named ultra-early ICH score, containing admission GCS score (3–4: 2 points; 5–12: 1point), baseline ICH volume ˃ 30 ml (1 point), positive IVH (1 point), infratentorial hemorrhage (1 point), age ≥ 80 (1 point), positive BS (1 point), positive BHS (1 point), and positive IS (1 point). Their results showed that the ultra-early ICH score was a useful clinical assessment tool for risk stratification concerning functional outcomes and provided guidance in clinical decision-making in acute ICH.

Besides, Brouwers et al. (19) proposed a 9-point prediction score that included warfarin use (no [0 point] or yes [2 points]), a shorter time to CT (> 6 h [0 point] or ≤ 6 h [2 points]), CTA SpS (absent [0 point], present [3 points], or unavailable [1 point]), and baseline ICH volume (< 30 ml [0 point], 30-60 ml [1 point], or >60 ml [2 points]). Based on the 9-point prediction score, Huynh et al. (95) proposed a derivation of the PREDICT A/B scores. Number of SpS, time from onset, warfarin use or an international normalized ratio > 1.5, GCS, and NIHSScale were included in the PREDICT A/B scores. PREDICT A showed improved discrimination compared with a 9-point prediction score, but independent validation was required, whereas the performance of PREDICT B varied by definition of HE.

The TRAIGE trial was conducted across 10 stroke centers in China as a randomized, placebo-controlled study aimed at assessing the efficacy of tranexamic acid in preventing acute intracerebral haemorrhage growth (95). Eligible patients were identified using imaging markers such as SpS, BHS, or BS on CT or CTA, and had to be treatable within 8 h of symptom onset. Participants were randomly assigned to receive either tranexamic acid or a placebo in a 1:1 ratio. However, the study results revealed that tranexamic acid did not significantly prevent intracerebral haemorrhage growth among patients at risk of HE and treated within 8 h of stroke onset. As a result, larger studies are necessary to gain a better understanding of the effectiveness of tranexamic acid. Notably, this trial underscores the potential utility of imaging markers in identifying eligible patients for study participation.

Based on the studies mentioned above, we recommend that clinicians use comprehensive scoring systems to identify high-risk HE patients. When only NCCT is available, we suggest using the BAT score. Another option is the 7-point prediction score proposed by Huang et al. (21). These scoring systems can assist clinicians in poor medical institutions to quickly identify high-risk HE patients and conduct appropriate anti-expansion therapy. When CTA is available, we suggest using the PREDICT A/Bscores identify ICH patients with high-risk HE. We summarized the different prediction scores associated with HE and clinical outcomes in ICH in Table 2.

**Table 2:** Summary of different prediction scores associated with HE and clinical outcomes in ICH.

Author	Scores	Year	Nation	Study Design	Paticipants (*n*)	Components of the scores	Primary outcome	Categorized score			
								0	1–3	4–9	
Brouwers et al. (19)	9-point score	2014	U.S.	Prospective cohort Multi-center	1,012	Warfarin sodium use (No: 0; Yes: 2)	HE (%)	5.7	12.4	36.4	
						Time to initial CT (h; ≤6: 2; ˃6: 0)	In-hospital mortality (%)	2.9	17.8	23.2	
						Baseline ICH volume (ml; <30: 0; 30–60: 1; ˃60: 2)					
						CTA spot sign (Absent: 0; Present: 3; Unavailable: 1)	3-month mortality (%)	5.7	23.2	50.2	
						Total 0–9					
Huynh et al. (95)	PREDICT A score	2015	Canada	Retrospective PREDICT study	301	GCS score (14–15: 0; ≤13: 4)	HE (%)	0–2		15–23	
						Hours from onset to CT (h; ≤1: 5; 1–2: 4; ˃2–3: 3; ˃3–4: 2; ˃4–5: 1; ˃5: 0)		7.1		70	
						Warfarin use or INR >1.5 (No: 0; Yes: 4)					
						CTA spot sign number (0 spot: 0; 1 spot 4; ≥2 spots: 8)					
						Total 0–23					
	PREDICT B score					NIHSS score (0–4: 0; 5–14: 4; ≥15: 7)		0–5		21–28	
						Hours from onset to CT (h; ≤1: 5; ˃1–2: 4; ˃2–3: 3; ˃3–4: 2; ˃4–5: 1; ˃5: 0)		5.6		73.3	
						Warfarin use or INR >1.5 (No: 0; Yes: 7)					
						CTA spot sign number (0 spot: 0; 1 spot 4; ≥2 spots: 9)					
						Total 0–28					
Morotti et al. (92)	BAT score	2018	Italy	Retrospective Multi-center	1,539	Blend sign (Present: 1; Absent: 0)	HE (%)	< 3		≥ 3	
						Any hypodensity (Present: 2; Absent: 0)		11.0		50.8	
						Time from onset to CT (h; <2.5: 2; ≥2.5 or unknown: 0)					
						Total 0–5					
Fu et al. (93)	10-Point Score	2020	China	Retrospective single center	216	Time to initial CT scan ≤3 h: 2	HE (%)	≥ 3			
						Hematoma volume > 30 ml: 1		SEN	SPE	PPV	NPV
						Island sign:6		97.8	92.7	90.9	98.3
						Black hole sign:1					
						Total 0–10					
Yang et al. (94)	uICH score	2021	China	Retrospective single center	310	Admission GCS score (3–4: 2; 5–12: 1; 13–14: 2)	Poor outcome	AUC	SEN (%)	SPE (%)	Optimal points
						Baseline ICH volume (ml; <30: 0; ≥30: 1)		0.85 (0.80–0.89)	71.7	84.2	2
						Presence of intraventricular hemorrhage (Present: 1; Absent: 0)					
						Infratentorial hemorrhage (Present: 1; Absent: 0)	30-day mortality	0.86 (0.80–0.91)	87.2	69.7	2
						Age (y; < 0: 0; ≥80: 1)					
						Blend sign (Present: 1; Absent: 0)					
						Black hole sign (Present: 1; Absent: 0)	90-day mortality	0.nnnnnn(0.81–0.92)	84.7	73.7	2
						Island sign (Present: 1; Absent: 0)					
						Total 0–9					
Huang et al. (21)	Grading system	2018	China	Retrospective single center	266	Hours from onset to CT (h; ≤6: 1; 6–24: 0)	HE (%)	0	1–3	4–7	
						Baseline ICH volume (ml; <30: 0; ≥30: 1)		3.45	34.48	76.47	
						Blend sign (Present: 1; Absent: 0)					
						Island sign (Present: 1; Absent: 0)					
						Swirl sign (Present: 1; Absent: 0)					
						Anticoagulant use or an INR > 1.5 (No: 0; Yes: 2)					
						Presence of intraventricular hemorrhage (Present: 1; Absent: 0)					
						Total 0–7					

In summary, the approach of stratifying high-risk HE patients based on imaging markers and patient clinical information is interesting. Comprehensive scoring systems can assist clinicians in different medical institutions to quickly identify high-risk HE patients and conduct appropriate anti-expansion therapy. This is a crucial point to improve poor outcomes, disability, and mortality rates.

## 9. Challenges and areas of focus for the future

In accordance with baseline hematoma size and location, HE is regarded an independent predictor of prognosis and a prospective target for acute-phase therapy of ICH. In clinical practice, regular monitoring of imaging indicators can identify patients who require anti-expansion therapy. Nevertheless, it is unclear if anti-expansion therapy improves clinical functional result or survival, which is a critical subject for further research. The Antihypertensive Therapy of Acute Cerebral Hemorrhage (ATACH-2) study found that aggressive blood pressure management did not reduce mortality or disability (96). Yet, the hemostatic medication Factor VII’s capacity to diminish HE was most potent during the first 2.5 h, as demonstrated in the Factor VII for Acute Hemorrhagic Stroke Trial (FAST) experiment (97, 98). A subsequent analysis of the ATACH-2 study found that reducing blood pressure ultra-early lowers HE and improves outcomes in people with ICH (99).

Recent findings from the Minimally Invasive Surgery Plus Alteplase for Intracerebral Hemorrhage Evacuation (MISTIE) III study showed that a minimally invasive technique was safe for patients with ICH. Nevertheless, it demonstrated no effect in terms of the primary outcome in a subset of individuals. Patients with low risk of HE may be better suited for MIS methods when they become available because to their decreased risk of postoperative rebleeding (100). The BS and BHS were linked to postoperative rebleeding in patients with ICH after minimally invasive surgery, according to retrospective investigations (101, 102). Despite this, individuals with consistently formed hematomas had satisfactory functional results following surgery (103).

Newly suggested recommendations for identifying, reporting, and interpreting these radiological indicators may give more proof of imaging markers’ predictive efficacy (104). As a result, future initiatives should aim to improve the standardization of the ever-expanding vocabulary for HE imaging indicators and determine if they should be utilized to select patients for ICH clinical trials. Future imaging research in ICH should focus on developing user-friendly systems that include imaging markers and parameters. When artificial intelligence is integrated into the clinical workflow as a tool to aid clinicians, more accurate radiological assessments can be performed (105–108).

## 10. Conclusion

The management of ICH presents a significant challenge, and identifying high-risk patients for HE is crucial to improving outcomes. The use of imaging markers for HE prediction can aid in the rapid identification of such patients and may serve as potential targets for anti-HE therapies in the acute phase of ICH. Therefore, further research is needed to establish the reliability and validity of these markers in identifying high-risk patients and guiding appropriate treatment decisions.

## Author contributions

Y-WH and H-LH developed the initial idea for this study, contributed to the original draft, and contributed equally and are co-first authors. Z-PL and X-SY searches the relevant references and was responsible for the revision of the draft. Y-WH created the figure of etiology. X-SY provided the funding. All authors contributed to the article and approved the submitted version.

## Funding

This study was funded by the Project of Mianyang Central Hospital (2021YJ006).

## Conflict of interest

The authors declare that the research was conducted in the absence of any commercial or financial relationships that could be construed as a potential conflict of interest.

## Publisher’s note

All claims expressed in this article are solely those of the authors and do not necessarily represent those of their affiliated organizations, or those of the publisher, the editors and the reviewers. Any product that may be evaluated in this article, or claim that may be made by its manufacturer, is not guaranteed or endorsed by the publisher.

## Figures and Tables

**Table:** 

ICH	Intracerebral hemorrhage
HE	Hematoma expansion
CT	Computed tomography
CTA	Computed tomographic angiography
MRI	magnetic resonance imaging
SpS	Spot sign
LS	Leakage sign
STS	Spot-tail sign
IS	Island sign
SaS	Satellite sign
IoS	Iodine sign
BS	Blend sign
SwS	Swirl sign
BHS	Black hole sign
FBL	Fluid-blood level
SAHE	Subarachnoid Extension
IVH	Intraventricular hemorrhage
NCCT	Noncontrast computed tomography
HU	Hounsifield unit
MCTAV	Minimal computed tomography attenuation value
ATACH-2	the antihypertensive treatment of acute cerebral hemorrhage
FAST	The factor VII for acute hemorrhagic stroke trial
MISTIE	The minimally invasive surgery plus alteplase for intracerebral hemorrhage evacuation
APOE	Apolipoprotein E
BBB	Blood–brain barrier
SEN	Sensitivity
SPE	Specificity
PPV	Positive predictive value
NPV	Negative predictive value
GSI	Gemstone spectral imaging
IC	Iodine concentration
CAA	Cerebral amyloid angiopathy
TRAIGE	Tranexamic acid for acute ICH growth prEdicted by spot sign
